# Non-Breeding Eusocial Mole-Rats Produce Viable Sperm—Spermiogram and Functional Testicular Morphology of *Fukomys anselli*

**DOI:** 10.1371/journal.pone.0150112

**Published:** 2016-03-02

**Authors:** Angelica Garcia Montero, Christiane Vole, Hynek Burda, Erich Pascal Malkemper, Susanne Holtze, Michaela Morhart, Joseph Saragusty, Thomas B. Hildebrandt, Sabine Begall

**Affiliations:** 1 Department of General Zoology, Faculty for Biology, University of Duisburg-Essen, Essen, Germany; 2 Department of Game Management and Wildlife Biology, Faculty of Forestry and Wood Sciences, Czech University of Life Sciences, Prague, Czech Republic; 3 Leibniz Institute for Zoo and Wildlife Research, Reproduction Management, Berlin, Germany; Zhejiang University, CHINA

## Abstract

Ansell’s mole-rats (*Fukomys anselli*) are subterranean rodents living in families composed of about 20 members with a single breeding pair and their non-breeding offspring. Most of them remain with their parents for their lifetime and help to maintain and defend the natal burrow system, forage, and care for younger siblings. Since incest avoidance is based on individual recognition (and not on social suppression) we expect that non-breeders produce viable sperm spontaneously. We compared the sperm of breeding and non-breeding males, obtained by electroejaculation and found no significant differences in sperm parameters between both groups. Here, we used electroejaculation to obtain semen for the first time in a subterranean mammal. Spermiogram analysis revealed no significant differences in sperm parameters between breeders and non-breeders. We found significantly larger testes (measured on autopsies and on living animals per ultrasonography) of breeders compared to non-breeders (with body mass having a significant effect). There were no marked histological differences between breeding and non-breeding males, and the relative area occupied by Leydig cells and seminiferous tubules on histological sections, respectively, was not significantly different between both groups. The seminiferous epithelium and to a lesser degree the interstitial testicular tissue are characterized by lesions (vacuolar degenerations), however, this feature does not hinder fertilization even in advanced stages of life. The continuous production of viable sperm also in sexually abstinent non-breeders might be best understood in light of the mating and social system of *Fukomys anselli*, and the potential to found a new family following an unpredictable and rare encounter with an unfamiliar female (“provoked or induced dispersal”). Apparently, the non-breeders do not reproduce because they do not copulate but not because they would be physiologically infertile. The significantly increased testes volume of breeding males (compared to non-breeders) is in agreement with previously found higher testosterone levels of breeders.

## Introduction

African mole-rats (family Bathyergidae) are subterranean rodents occurring in sub-Saharan Africa, comprising six genera with highly divergent social and mating systems. One of these genera, the *Fukomys* mole-rat, is highly social (eusocial), living in families of an average size of twelve, where only a single pair reproduces (so called breeders) while most of the offspring remain for most of their lives in the natal nest (i.e. non-breeders). The non-breeding animals help to extend, maintain and defend the common burrow system, which may be up to 3 km long [1–4].

The Ansell's mole-rat (*Fukomys anselli*) from Zambia is a typical representative of the genus, the reproductive and social biology of which was intensively studied both in the laboratory [5–9] and in the field [3, 10]. The family members recognize each other individually and incestuous mating is avoided [7], a situation corresponding thus to Westermarck's effect. The breeding pair is sexually very active and mating can be observed every day all the year round (non-seasonal breeders). The non-breeding animals are sexually abstinent within their respective natal families, but if encountering an unfamiliar partner of opposite sex outside the family they begin almost immediately to mate [2, 5, 7, 9]. It is not yet quite clear how often, under which conditions, and at which age Ansell's mole-rats tend to disperse and how new families are founded in the field. However, it is reasonable to assume, and the above cited laboratory studies suggest it, that dispersal (i.e. leaving the family and founding own family) is induced (provoked) by incidental encounter with an unfamiliar adult mole-rat of opposite sex (cf. also [2, 11]). This can happen e.g. at margins of extended partially overlapping burrow systems.

Since their sexual activity is inhibited purely on a cognitive basis [2, 5, 7, 9], we might expect that spermatogenesis takes place spontaneously even in sexually abstinent males similar to the situation in humans. The aim of this study is to test this hypothesis.

Thus far, diverse aspects of male breeding physiology were studied in several species of African mole-rats. Non-breeding male naked mole-rats (*Heterocephalus glaber*) had lower testosterone concentration compared to the breeders [12–14]. Almost all breeding naked mole-rats, but only two out of seven non-breeding males possessed motile spermatozoa [15] (Saragusty et al., unpublished data). The sexual inactivity, and apparently also sterility, of "workers" in at least larger colonies of the naked mole-rat is based on social suppression by the breeding female [12] rather than cognition-based incest avoidance. This physiological restraint of reproduction can be reversed when a non-breeding animal emancipates from its colony: after 5–8 days the concentration of the sexual hormones begins to rise and the animals become potentially reproductively active [15].

In the Damaraland mole-rat (*Fukomys damarensis*), a species of hairy eusocial mole-rats, which lives like the Ansell's mole-rat, in families that are smaller than in the naked mole-rat and in which incest avoidance is also the main mechanism of reproductive control [16, 17], the relative testis weight in breeding males was higher than in non-breeding males, but the number of motile spermatozoa in epididymis did not differ significantly between the two groups [15]. Thus far, no comparative data on spermatogenesis are available for the Ansell's mole-rat. De Bruin et al. [18] found higher plasma testosterone levels and larger diameter of the seminiferous tubules in breeding compared to non-breeding males of Ansell’s mole rats. The mean testes volume and mean relative testes mass showed no significant difference between both groups.

In all the above mentioned studies on male mole rat breeding biology, sperm was obtained by autopsies or biopsies from the epididymis, and production and parameters of seminal fluids (ejaculate, semen) were not investigated and not considered. It is clear, however, that important fertility parameters (such as ejaculate volume, number of ejaculated sperm, their motility and viability) remain unnoticed by this method of obtaining. Therefore we decided to study parameters of more ‘natural’ ejaculate and used electroejaculation as a method of choice to obtain it. This method is, moreover, less invasive than a biopsy and, compared to autopsies, spares lives of animals (cf. [19–22]).

Beside of introducing electroejaculation as a new method to obtain semen in a subterranean mammal, we will furthermore compare spermiogram parameters, testicular histology and volume of reproductively active (breeders) and non-reproducing (non-breeders) males of the Ansell’s mole-rat. Based on the sexual behavior of non-breeders when being allowed to mate, we expect no significant differences in the measured parameters between the two male groups.

## Material and Methods

### Ethics statement

All procedures involving wild-caught animals were performed in a human manner and were approved by the Zambia Wildlife Authority (ZAWA) and the Institutional Animal Care and Use Committee at University of South Bohemia and Ministry of Education, Youth and Sports (n. 12935/2007/30). The official permit n. 223623 approving the field work on the Ansell’s mole- rat in Zambia was issued by ZAWA. The field work (trapping) was conducted in the Lusaka East Forest Reserve in Zambia from April to July 2010. Trapping permission was issued by the Department of Forestry, Lusaka. Mole-rats were captured using Hickman traps set close to clusters of fresh molehills.

The laboratory experiments were approved by the Landesamt für Natur, Umwelt und Verbraucherschutz Nordrhein-Westfalen, reference number 84–02.05.20.12.145. All animals used in this study fully recovered from anaesthesia and electroejaculation, and no complications occurred during or after the treatment.

### Animals

We examined altogether 20 adult male Ansell's mole-rats (*Fukomys anselli*): seven breeders and 13 non-breeders from twelve different families. The numbers of animals used for each of the specific examinations are given below. Most animals were born in captivity. Two of the breeders were captured in Lusaka, Zambia, in 2010. All animals were older than 12 months and thus expectedly sexually mature. Average age and body mass at the time of investigation are given in Table 1.

**Table 1:** Main parameters (mean, SD given in parenthesis) of male reproductive characteristics measured in Ansell’s mole-rats. Volumes estimated for the right and left testis of each male were averaged to obtain mean absolute and relative testes volumes, respectively. *The minimum age at the time of testis measurement was estimated for two specimens since they were caught in the field in Lusaka, Zambia.

status	age* (days)	body mass (g)	absolute testes volume (mm^3^)	relative testes volume (mm^3^)	ejaculate volume (μl)	concentration (x10^6^/ml)	progressive motility (%)	vitality (%)
**breeder**	2866	94.7	90.5	0.94	2.1	191.3	48.8	80.0
	(1411)	(23.0)	(32.7)	(0.14)	(1.04)	(189.9)	(25.7)	(12.6)
	n = 7	n = 7	n = 7	n = 7	n = 4	n = 4	n = 4	n = 4
**non-breeder**	1836	126.8	65.5	0.5	3.5	183.6	52.3	79.0
	(805)	(32.9)	(33.9)	(0.22)	(1.3)	(93.2)	(18.5)	(11.7)
	n = 13	n = 13	n = 13	n = 13	n = 4	n = 4	n = 4	n = 4

The animals were kept at the animal facilities of the Faculty of Biology of the University Duisburg-Essen in Essen, Germany. Room temperature was 25 ± 1.0°C with 40–50% humidity and artificial light conditions (12 h D:12 h L). The mole-rats were housed in glass terraria (size being dependent on family size) filled with horticultural peat to a depth of about 3–5 cm. Tissue paper was provided as nest material. Animals were fed with carrots and potatoes ad libitum, while apples, lettuce and cereals were provided once per week.

### Anaesthesia

For examination, the mole-rats were anaesthetized using a single intramuscular dose of 6 mg/kg ketamine and 2.5 mg/kg xylazine (Ceva, Düsseldorf, Germany) [23]. After losing the righting reflex through chemical restraint the animals were placed in dorsal position and the hind limbs were spread and hand-fixed. The abdominal and pelvic area was depilated by hair removal cream (Veet by Reckitt Benckiser, Heidelberg, Germany) to enable ultrasonographic examination.

### Testes size

Length, breadth, and width of both testes were measured in 13 anaesthetized males by means of ultrasonic imaging (Vevo 2100; VisualSonics Inc., Toronto, Canada). The same parameters were measured in seven autopsied males directly by means of a digital caliper. Volume of testes was estimated from the measured dimensions as an ellipsoid volume

$$\text{V }=\;\frac{1}{6}\cdot \;\pi \;\cdot \text{ a }\cdot \text{ b }\cdot \text{ c}$$

with a = length, b = breadth, and c = width. For each male, absolute testes volume and testes volume relative to body mass were averaged over left and right testes.

### Electroejaculation

Eight spermiograms were established taking one semen sample per individual (four non-breeding and four breeding males) of six different families. The penis was thoroughly cleaned with tissue paper. Semen was collected by electroejaculation (Seager model 14, Dalzell USA Medical Systems, The Plains, VA, USA) using a specially designed miniature electrical probe inserted approximately 1.5 cm into the anus.

The prostate was stimulated in intervals (stimulations and pauses alternated) starting at 2 V. The voltage was increased at each stimulation phase by approximately 0.5–1 V until the animal ejaculated (maximum voltage applied: 9 V, 15 stimulations). The total duration of the interval stimulation (including pauses) ranged between 96 seconds and 8:30 minutes.

Ejaculated semen was collected from the urethra orifice at the tip of the erected penis with a hand-held pipette and immediately diluted into 20 μL of pre-warmed (37°C) Medium 199 (M7528, Sigma-Aldrich Chemie GmbH, Taufkirchen, Germany).

### Semen evaluation / Spermiogram

The volume of the ejaculated semen was measured by means of an automatic pipette (0–10 μl). Sperm motility was analyzed using a computer-aided sperm analysis (CASA) system (AndroVision, Minitube, Tiefenbach, Germany). Samples (5 μL) were loaded onto pre-warmed (37°C) Makler counting chamber (Sefi Medical Instruments, Haifa, Israel) and analyzed by the CASA system, which consisted of an optical phase-contrast microscope (CX41; Olympus, Tokyo, Japan) with a warm stage (37°C) and a digital camera (avA1000-100gc, Basler, Ahrensburg, Germany). Images were captured and analyzed using AndroVision software (Minitube, Tiefenbach, Germany). Analysis was carried out using a 10X negative phase contrast objective (PLN10XPH; Olympus, Tokyo, Japan). Data on total and progressive sperm motility was acquired. Software acquisition settings were set at a rate of 60 frames per second (60 Hz) with 30 frames acquired per field. A total of eight randomly-selected fields were acquired for each sample. Sperm were considered immotile when the average orientation change (AOC) of the head was under 9.5°. Motility was considered stationary when AOC ≥ 9.5° and the distance in straight line (DSL) was under 6 μm. Motility was progressive when AOC ≥ 9.5° and DSL ≥ 6 μm.

Sperm viability was assessed by mixing 10 μl of the previously prepared semen suspension with 10 μl of eosin-nigrosin solution (eosin Y yellow CI 45380, nigrosin CI 50420 dissolved in 0.9% NaCl; VWR International, Darmstadt, Germany), incubated at room temperature for 2 min and then preparing smears that were air-dried before evaluation. At least 100 spermatozoa of each sample were examined under a light microscope (CX41; Olympus, Tokyo, Japan, oil immersion; 1000x). White (unstained) spermatozoa were classified as viable and those that showed pink or red coloration were classified as dead.

Sperm aliquots were also fixed in Hancock’s solution (4% formol citrat) and assessed for acrosome integrity and sperm morphology [24, 25]. At least 100 spermatozoa were evaluated by phase-contrast microscopy (CX41; Olympus, Tokyo, Japan, oil immersion; 1000x). The acrosome was first evaluated according to the following categories: normal (intact acrosome), swollen, detaching, and detached (collectively referred to as non-intact acrosome). One hundred spermatozoa with intact acrosome were then assessed for morphology and were classified as normal, or as having a defect in the head, neck, midpiece, or tail. Sperm morphology included search for a wide range of abnormalities as previously described [25]. In case one single sperm showed several malformations each was noted separately, but the sperm was counted as one single cell.

### Histological analysis of testes

Testes of six Ansell’s mole-rats (three breeders, three non-breeders) that had been sacrificed and perfused within the framework of other projects with 4% paraformaldehyde (PFA) in 0.1 M phosphate buffer (PB) and postfixed overnight were used for histological examination. The testes were dehydrated in an ascending series of ethanol, cleared in xylene and embedded in paraffin using an automated spin tissue processor (Microm STP 120) and an embedding station (Leica EG 1160). Sections of 8 μm thickness were cut on a rotary microtome (Microm HM 340E) and stained with nuclear fast red and aniline blue-orange G (modified azan-staining after Geidies, [26]. Views of selected sections were digitalized with a light microscope (Olympus BX40) at 200x magnification and subsequently analyzed in ImageJ (v. 1.47v) [27]. From each section, three randomly chosen regions (area of 0.372 mm²) were analyzed and the percentage of the total area analyzed occupied by Leydig cells and seminiferous tubules determined. The mean calculated over the obtained values from the three areas of each section was then used in further analyses. On average, seven sections per testis were analyzed and for most animals measurements were made on both testes and averaged afterwards. We also measured the mean diameters of seminiferous tubules. Only perpendicular cross-sections apparent as circular tubules (ratio between max. height to max. width > 0.9) were taken into account.

### Statistical analysis

All means are given as x±SD (mean ± standard deviation). All samples were normally distributed as assessed by visual inspection of Q-Q-plots and Shapiro-Wilk test (p>0.05). Samples for non-breeding and breeding males showed comparable levels of variance (Levene-test; p>0.05). We used Pearson’s correlation coefficient as a measure for a linear correlation between age and body mass. Mean values for age and body mass calculated for non-breeding and breeding males were compared by unpaired two-sample t-tests. A paired t-test was applied to compare, respectively, the absolute and relative volume of the left and right testes of the same males. We used general linear model (GLM) ANCOVA with age and body mass as continuous variables and reproductive status as a factor (multiplicative model) and dropped stepwise non-significant variables (based on AIC, Akaike information criterion) to find the linear regression model that fits best the data (dependent variables were testes volume, ejaculate volume, total number of sperms, vitality, and motility, respectively). We are aware that small data sets for ejaculate volume, total number of sperms, vitality, and motility restricts the application of parametric tests, but as it is important to control for body mass we had to use ANCOVA. In addition, we used the Mann-Whitney-U-test (MW U-test) to compare differences between breeders and non-breeders in these parameters. These tests revealed similar results. Differences between breeders and non-breeders in the relative area occupied by seminiferous tubules and by Leydig cells, respectively, and in the diameter of the seminiferous tubules have been compared by means of MW U-test. All statistical analyses were performed in R [28].

## Results

### Age, body mass, and testes size

Breeding males (age: 7.9±3.9 years, n = 7) were older than non-breeding males (age: 5.0±2.2 years, n = 13), the difference being marginally non-significant (Table 1, *T* = 2.094, *P* = 0.0501). Non-breeding males were significantly (*T* = -2.218, *P* = 0.034) heavier (126.8±32.9 g, n = 13) than breeding males (94.7±23 g, n = 7). Age and body mass were not correlated in the current sample (Pearson’s correlation coefficient, *R* = -0.05).

**Fig 1:**
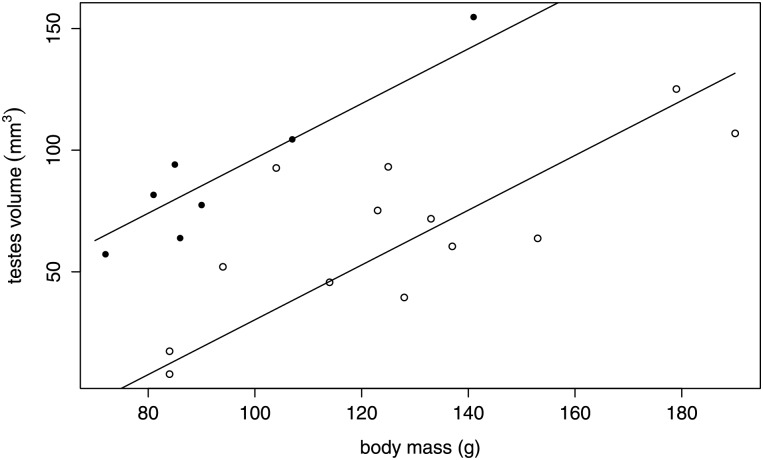
Reproductive males have larger testes compared to non-reproductive males. Absolute testes volume (mm^3^) relative to body mass (g) for breeders (filled circles) compared to non-breeders (open circles) of Ansell’s mole-rats (*Fukomys anselli*).

**Fig 2:**
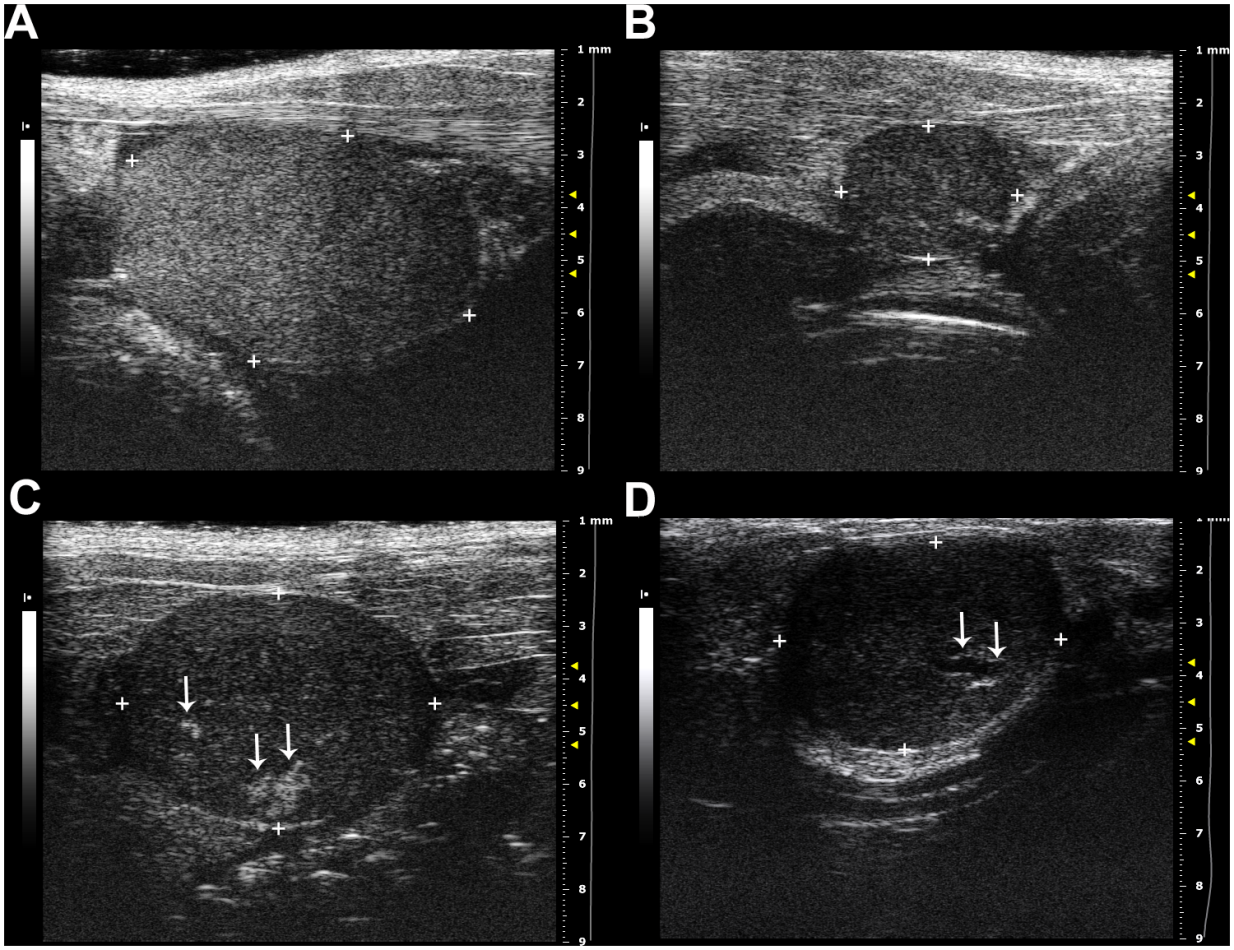
Ultrasonographic appearance of *Fukomys anselli* testicles. Testicles of breeders (A) compared to non-breeders (B) were significantly larger. Irregular hyperechoic lesions were found in 10 out of 13 individuals (C); additionally cystic alterations were present in two out of 13 individuals (D).

Absolute and relative volumes of the left and right testes were not significantly different (paired t-tests, *P*>0.05, n = 20). Relative testes volume of breeders (0.94±0.14 mm^3^) was significantly larger than in non-breeders (0.5±0.22 mm^3^) (t-test, *T* = 4.762, *P*<0.0001, Table 1, Figs 1, 2A and 2B). Mean absolute testes volumes of breeders and non-breeders were 90.5±32.7 mm^3^ and 65.5±33.9 mm^3^, respectively (Table 1). Based on GLM ANCOVA, absolute testes volumes (means of left and right testes) could be best fitted with body mass (*F* = 29.9, *P*<0.0001) and reproduction status (*F* = 23.5, *P*<0.001) as explaining variables (Fig 1). Age had no significant effect on testes volumes (*F* = 0.27, *P* = 0.61). Well-defined small (up to 0.89 mm x 0.68 mm) multiple non-shadowing and hyperechoic lesions of irregular shape were found during ultrasound examination in the testicles of 10 out of 13 individuals (Fig 2C). In two individuals, additionally, cystic alterations (up to 1.22 mm x 0.33 mm) were present in one of the testicles (Fig 2D).

### Sperm analysis

Both groups, breeders (n = 4) and non-breeders (n = 4), reacted to the electric stimulation, and an erection could be observed for longer penises (i.e. an erection might be present in smaller penises as well, but it was inconspicuous). All individuals produced an ejaculate, thus, the success rate of the electroejaculation was 100% in both groups.

Some of the animals urinated before they ejaculated possibly because of the muscle relaxing effect of xylazine [19] or due to the effect of the electric stimulus on the urinary bladder. The urine was easy to distinguish from the ejaculate given the higher volume and clear appearance, compared to the turbid appearance of the ejaculate.

We started to collect the sample as soon as a droplet of turbid fluid was seen at the tip of the penis (probably the “pre-ejaculate”). Average time until appearance of the pre-ejaculate was 139±153 sec. Usually, a short time later at 251±160 sec, a second, higher amount of semen was collected (ejaculate). The time between pre-ejaculate and ejaculate varied individually (mean 113±74 sec).

The mean volume of the ejaculate for both groups was 3.8±1.98 μL. Mean ejaculate volume in breeders was 2.1±1.04 μL and in non-breeders 3.5±1.29 μL. This difference was, however, not significant (ANCOVA, F = 2.947, *P* = 0.137, MW-U-test, *U* = 7.5, *P* = 0.886).

The total amount of sperms (mean sperm concentration per μL multiplied by sperm volume) for both groups was 187.4 x10^6^±138.5 x10^6^ sperms/mL and did not deviate significantly (ANCOVA, F = 0.0054, *P* = 0.944, MW-U-test, *U* = 7, *P* = 0.773) between breeders (191.3 x10^6^±189.9 x10^6^ sperms/mL) and non-breeders (183.6x10^6^±93.25 x10^6^ sperms/mL).

The viability analysis showed 79.5±11.3% live sperm at the time of ejaculation. The viability of the sperm of breeders (80±12.7%) and of non-breeders (79±11.7%) was not significantly different (ANCOVA, F = 0.0134, *P* = 0.912, MW-U-test, *U* = 8, *P* = 1).

The progressive motility was 50.5±20.8%. The progressive motility of sperms produced by breeders (48.8±25.7%) and non-breeders (52.3±18.5%) did not show significant deviations (ANCOVA, F = 0.0489, *P* = 0.832, MW-U-test, *U* = 7.5, *P* = 0.886).

### Sperm morphology

**Fig 3:**
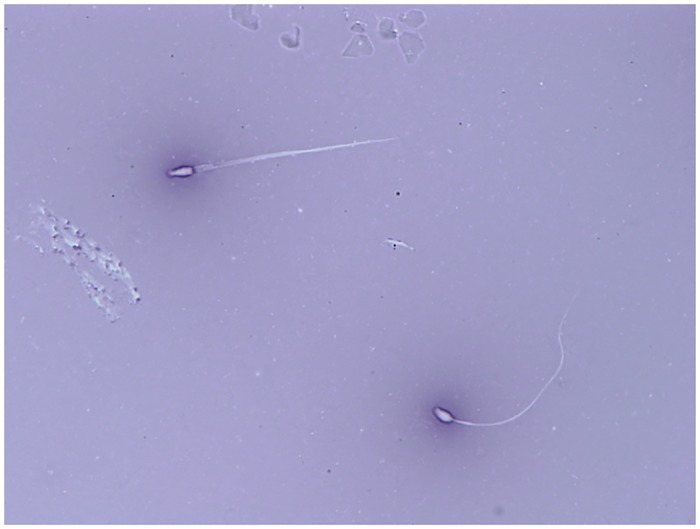
Normal morphology of Ansell’s mole-rat sperm. The samples were obtained by electroejaculation (light microscopy, magnification 600x, oil immersion, eosin & nigrosin staining).

The sperm head in *F*. *anselli* is oval from the frontal point of view. From lateral view, the acrosome is more prominent, giving the head the form of a pear. The midpiece is longer than the head and thicker than the tail (Fig 3). There are no significant differences in sperm morphology between breeders and non-breeders. The percentage of normal sperms was 86±0.3%. The most common abnormality was a sperm-head-deformity and the second most frequent abnormality was a deformity of the sperm tail. The abnormalities of the midpiece affected only a few of the sperm.

### Histological analysis

**Fig 4:**
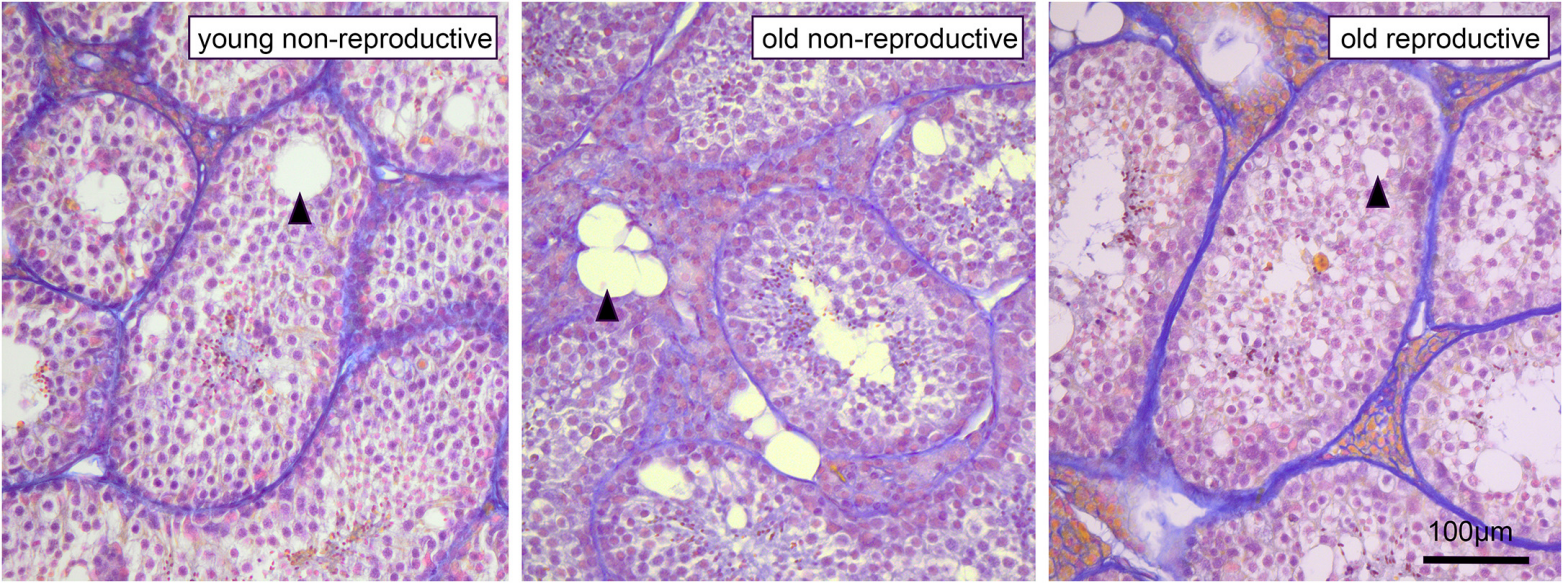
Light microscopy (x200) of histological testicular sections (8μm thickness) of three Ansell's mole-rats. A. young non-reproductive (age: 22 months), B. old non-reproductive (age: 8.7 years), C. reproductive male (age: 9.8 years). The germinal epithelium of the seminiferous tubules and to a lesser degree the interstitial testicular tissue of all individuals were characterized by lesions (vacuolar degenerations) exemplarily indicated by black arrowheads.

The histology of the testis revealed no difference between breeders and non-breeders in terms of the relative area (percentage) occupied by Leydig cells (breeders: 6±1.7%, n = 3; non-breeders: 5.2±0.9%, n = 3) and seminiferous tubules (breeders: 67±2.7%; non-breeders: 71.4±3.3%) (MW-U-test, *P*>0.05). The mean diameter of single seminiferous tubules does not differ between breeders (171±24 mm, n = 3) and non-breeders (152±17 mm, n = 3) (MW-U-test, *P* = 0.2). Histological microscopy revealed empty (i. e. relatively spermatozoa-free) lumina of the seminiferous tubules and conspicuous lesions (“vacuoles”) of the seminiferous epithelium and partly of the interstitial testicular tissue (Fig 4), however, there were no significant differences in this aspect between non-breeders (Fig 4A and 4B) and breeders (Fig 4C).

## Discussion

We found no qualitative differences in the histological structure of the testes, in sperm morphology, ejaculate volume, total number of sperms and sperm motility between breeders and non-breeders of Ansell’s mole-rats. The functional relevance of the frequently encountered testicular lesions is not clear, as they neither consistently correlate with age, social status, nor with sperm quality. The only quantitative difference refers to the volume of the testes, which is significantly larger in breeders than in non-breeders.

### Spermiogram

The 100% success rate of electroejaculation shows that this method is suitable to obtain semen from Ansell’s mole-rats. As far as we know, this is the first report of electroejaculation in a subterranean mammal. Sperm analysis in other species such as the naked mole-rat or the Damaraland mole-rat were performed with sperm obtained directly from the epididymis by gonadectomy [15] and those results cannot be completely compared with the analysis of full ejaculate obtained by means of electroejaculation. Assuming that the anaesthesia has no influence on the spermiogram quality, we can compare spermiograms of mole-rats with those of other rodents studied. Sperm concentration of anaesthetized chinchillas (204x10^6^/ml) [20] is comparable to the sperm concentration found here in anaesthetized Ansell’s mole-rat (187x10^6^/ml). Mollineau et al. [21] used several different doses of ketamine and xylazine and two different induction times to find the best outcome of electroejaculation in agoutis (*Dasyprocta leporina*). The highest sperm concentration of 431x10^6^/ml was obtained under relatively high anaesthetic doses.

Spermiograms of Ansell’s mole-rats showed no conspicuous abnormalities, and a high percentage (approximately 85%) of normal sperm morphology indicates good quality. Non-breeding Ansell’s mole-rats produce sperm of the same quality as breeders. The genetic structure and average size of an Ansell’s mole-rat colony in the wild suggest that many non-reproductive animals do not find a partner and remain within their natal family [10]. Extra-pair paternities appear to be absent within the natal families, and 96.4% of the juveniles are sired by the dominant (founder) male of the colony [10]. Also in the laboratory, non-breeding males do not mate with female family members based on incest avoidance due to individual recognition [7, 29]. The spermatozoa produced by adult non-breeders staying in their natal family will probably undergo post-meiotic senescence and dissolute in the cauda epididymis [30] or get eliminated in urine [31]. The viable sperm in the epididymis of non-breeders thus needs to be renewed less frequently than the sperm regularly ejaculated by breeders who presumably produce sperm permanently (cf. [30]). Similarly, Faulkes et al. [5] found, comparable values of motile sperm (gained by epididymis autopsy) in *F*. *damarensis* breeders and non-breeders (but see Maswanganye [32]). In contrast to Ansell’s mole-rat, non-breeding males of the naked mole-rat (*Heterocephalus glaber*), another eusocial subterranean bathyergid, produce sperm with an atypical mammalian structure, with most spermatozoa classified as abnormal [33] (Saragusty et al. unpublished data).

Sperm morphology is rather variable in different rodent species. Roldan et al. [34] compared five different rodent groups (ctenomyids, murids, and diverse cricetine rodents) and found variations in sperm length and sperm head complexity ranging from simple sperms with a round/oval head to sperms with a head in the form of a hook (apical hook). The complexity of the spermatozoon’s head has been interpreted as a response to the selective pressure represented by cryptic female choice and sperm competition in polygynous species. The analysis of a much larger data set containing 226 mammalian species supported this hypothesis [35]. Furthermore, it was shown that sperm heads become longer and all sperm components increase with increasing level of sperm competition. However, although almost half of the species were rodents (n = 106), the data set comprised not a single bathyergid. Sperm of Ansell’s mole-rats is of a simple morphology with an pear-shape head shape and without conspicuous features like an apical hook. This is in line with the fact that sperm competition does not occur. Sperm of other subterranean mammals is also of similar morphology, as has been shown for e.g. breeding males of *Heterocephalus glaber* [33], *Spalax ehrenbergi* [36], and *Ctenomys talarum* [37]. This could be interpreted as an indication of absence of sperm competition.

### Testis volume and histology

In Ansell’s mole-rats, testicular volume relative to body mass was significantly higher in breeding males compared to non-breeding males. Beside the breeding status, body mass but not age explained much of the variation of absolute testis size, although age and body mass are positively correlated during the first postnatal year [8]. However, age and body mass were not correlated in the current sample, which might be based on the fact that we used only adult mole-rats who had already reached their adult (asymptotic) body mass. On the other hand, we observed that breeders older than about 3 years tend to decrease their body mass and be no more the largest individuals in their respective families. We described this phenomenon in females [38] and it is apparently also the case in males, though we have not studied it systematically. The larger relative and absolute testis size could be explained by either an increase of the testicular size of breeders or a decrease of the testicular size of non-breeders. Since non-breeders produce sperm of similar quality as breeders, it is unlikely that their testes degenerate. Breeders, on the other hand, ejaculate often, since copulation takes place regularly, non-seasonally, all year round, even during advanced stages of pregnancy or after delivery of pups, sometimes even several times per day [5, 7]. The increased sexual activity is obviously not directed to increase fertility but it might strengthen the bond of the reproductive pair. Large testes could help to sustain the constant production of sperm to ensure fertilization at times when the breeding female is receptive.

Previous experiments on male rats, rabbits and rams that were either ejaculated or sexually rested for different periods of time resulted in contradictory results: some studies demonstrated an increase in the size of the reproductive organs in response to the treatment, in other studies no significant differences could be shown [39–43] (see also Amann, unpublished, cited in Carson & Amann [44]). Beside of the fact that the testes size in rams and rabbits is also dependent on the season, the studied species are polygynous and large testes are conducive to sperm competition. Thus, even sexually rested rabbits or rats might sustain large testes for possible (future) sexual activity, especially since the sexual restraint is preposterous to nature for males of these species. The situation is, however, completely different in Ansell’s mole-rats where sperm competition does not occur due to monogamy. Here, most of the males (non-breeders) are never sexually active throughout their lives.

The larger testis volume of breeders is not simply due to an hyperplasia of *either* Leydig cells *or* expansion of seminiferous tubules, but of *both* components that proliferate or grow proportionally. Since the mean absolute diameter of seminiferous tubules is not significantly different in breeders and non-breeders, the proportional tubule area increase in breeders compared to non-breeders is based on its length, not on its diameter. We conclude that the absolute number of Leydig cells is higher and the seminiferous tubules are longer in the larger testis of breeders compared to smaller testes of non-breeders. On proximate terms, breeders are expected to have higher testosterone concentrations compared to non-breeders which has been indeed shown in the studied species [18] (but see our critical evaluation below) and as is also the case in naked mole-rats [13], but not in *F*. *damarensis* [45]. In naked mole-rats it has been revealed that up to 60% of the testicular tissue is made up by lipid rich interstitial cells, including Leydig cells [46, 47]. Faulkes et al. [13] speculate that the larger testes size in naked mole-rat breeders is caused by a higher amount of Leydig cells. Although not quantitatively determined, a visual inspection revealed larger numbers of Leydig cells in breeders compared to non-breeders [13].

Our finding that breeders have significantly larger relative testis volumes compared to non-breeders is in contrast to the study of de Bruin et al. [18] who did not find any difference in relative testicular mass or relative testicular volume between the two male groups. On the other hand, we did not find any significant differences in spermiogram parameters or histological testicular samples between reproductive and non-reproductive males, while de Bruin et al. [18] noticed significantly larger diameters of seminiferous tubules in breeders compared to non-breeders. Furthermore, in our study non-breeders were significantly heavier than breeders, while the opposite was the case in the study by de Bruin et al. [18]. The main difference between the two studies is the differentiation of the two male groups, i.e. breeders and non-breeders. While in our study the reproductively active males can be clearly assigned based on their sexual activity, de Bruin and colleagues identified breeders on the base of staining around the mouth and bulging abdominal testes. In our opinion, these features are far too weak and not categorical to allow an unambiguous classification. According to de Bruin et al. [18], reproductive males of Ansell’s mole-rats weigh on average 81 g and thus twice as much as non-reproductive males (on average 40 g). Additionally, they stress that the cohort of non-breeders in the body mass class 60–70 g is missing. This is in contrast to field data obtained by Šklíba et al. [3] who stated that “the largest individuals are usually breeding males followed by breeding females, but the difference in body mass of a breeding pair and the rest of the group was not always conspicuous and exceptions occurred”. Furthermore, de Bruin and colleagues used the body mass of colony members to assign the mole-rats to age classes based on the categorization of Sichilima et al. [48]: body mass of juveniles ranges between 3 and 24 g, sub-adults between 25 and 34 g, and adults have a body mass of more than 35 g. Previous studies on growth and development of Ansell’s mole-rats [5, 8] as well as live-trapping of mole-rats in Zambia and their further keeping in captivity (unpublished data) suggest that Ansell’s mole-rats with a body mass of 35 g (to 45 g) are definitively not grown-up. Thus, it is highly likely that de Bruin et al. [18] misclassified some of the subadult males (presumably non-breeders) in their study as adults. Furthermore, we suggest that the traditional claim that breeders are the largest individuals in a family is valid only for younger families, where the oldest offspring are not yet fully grown.

Interspecific comparisons of the size of testes are difficult because in some studies only absolute values and in other studies only relative values are given. Furthermore, some researchers measured only testicular volume and others weighed the testicular mass. Seasonal breeding of some bathyergids further impedes comparison. In *Georychus capensis* [49] and *Bathyergus suillus* [50] for instance, onset of the reproductive season is accompanied by an enlargement of testes. In the following, we will summarize some of the findings for different social bathyergids. Significantly larger absolute and/or relative testicular volume and/or testicular mass in breeders compared to non-breeders have been reported for several social bathyergids with reproductive skew: *Cryptomys hottentotus natalensis* during summer [51], *Fukomys damarensis* [15, 32], and *Heterocephalus glaber* [13, 15]. No significant difference in the testicular size between the two male groups has been found in *Cryptomys hottentotus hottentotus* [52] and *Cryptomys hottentotus natalensis* during the winter [51]. In contrast to Maswanganye et al. [32], Nice and colleagues [53] did not find a significant difference in the testicular mass between breeders and non-breeders of *Fukomys damarensis*. However, non-reproductive males of *F*. *damarensis* that were exposed to an unfamiliar female for 60 minutes had 27% heavier mass-corrected testes compared to reproductive males [53]. This example shows that increase in testes size can occur rapidly.

The lesions in the parenchyma of the seminiferous tubules resemble vacuolar degenerations reported for old [54–56], experimentally manipulated [57–59], or seasonally regressed testes [60–62] of mice, rats and other mammals including men. This degeneration is apparently a “normal” feature in *Fukomys* mole-rats (at least in *F*. *anselli*) and does not seriously hinder spermatogenesis and production of viable sperms. The sample size of here examined males is, however, too small to allow unambiguous quantitative evaluation and statistic correlation with age, phase of the life history, and sperm production (note that the histologically examined testes originated from other animals than those electroejaculated). The seminiferous tubules of naked mole-rats also show vacuolated degeneration [13, 47, 63–64], but its presence has not been discussed by the authors of the respective studies.

## Conclusion

Our results provide clear evidence that spermatogenesis in the non-breeders takes the normal course despite their sexual abstinence. In other words, the non-breeders do not reproduce because they do not copulate but not because they would be physiologically infertile. This is a comparable situation to that found in female non-breeders, who show normal oogenesis and folliculogenesis [38]. These anatomical findings are in agreement with behavioral observations [5–7, 9] showing that mating, including copulation with insertion in non-breeder Ansell’s mole-rats, can occur within seconds or just a few minutes after encounter of two unfamiliar mole-rats of opposite sexes. Furthermore, endocrinological data of female Ansell's mole-rats [65] support the thesis that non-breeders in in this species are physiologically fertile and ready for mating and reproducing if given an opportunity.
